# Cardiac sarcoidosis imaging features and latest advances in the management of arrhythmias: a case report

**DOI:** 10.1093/ehjcr/ytag147

**Published:** 2026-03-09

**Authors:** Rakesh Sharma, Ana Pericao, Mansimran Singh Dulay, Jonathan M Behar

**Affiliations:** Department of Cardiology, The Royal Brompton Hospital, Part of Guy’s and St Thomas’ NHS Foundation Trust, London SW3 6NP, UK; National Heart and Lung Institute, Imperial College, Guy Scadding Building, Cale Street, London SW3 6LY, UK; Department of Imaging Sciences, King's College London, London SE1 7EH, UK; Department of Cardiology, The Royal Brompton Hospital, Part of Guy’s and St Thomas’ NHS Foundation Trust, London SW3 6NP, UK; Department of Cardiology, The Royal Brompton Hospital, Part of Guy’s and St Thomas’ NHS Foundation Trust, London SW3 6NP, UK; Department of Cardiology, The Royal Brompton Hospital, Part of Guy’s and St Thomas’ NHS Foundation Trust, London SW3 6NP, UK; Department of Imaging Sciences, King's College London, London SE1 7EH, UK; Department of Cardiology, St Thomas’ Hospital, Part of Guy’s and St Thomas’ Hospital NHS Foundation Trust, London SE1 7EH, UK

**Keywords:** Cardiac sarcoidosis, Multimodality imaging, Ablation, Cardiac resynchronization therapy with defibrillator, Multidisciplinary team meeting, Case report

## Abstract

**Background:**

Cardiac sarcoidosis (CS) is a potentially life-threatening granulomatous inflammatory cardiomyopathy associated with conduction abnormalities, ventricular arrhythmias, and heart failure. Multimodality imaging plays a key role in diagnosis, risk stratification, and guiding therapy.

**Case summary:**

A 53-year-old male presented with an out-of-hospital ventricular fibrillation arrest in May 2022. Transthoracic echocardiography demonstrated moderate left ventricular (LV) systolic impairment with hypokinesis of the basal inferoseptum, anteroseptal, and basal to mid-inferior walls. Invasive coronary angiography excluded flow-limiting coronary artery disease, and a cardiac resynchronization therapy defibrillator was implanted.

Cardiac magnetic resonance imaging (CMR) was performed in February 2024, but image quality was suboptimal due to device artefact. It showed generalized hypokinesia with thinning and akinesia of the basal to mid-septum; late gadolinium enhancement was present but difficult to characterize. Over the following 18 months, serial CMR and ^18^F-fluorodeoxyglucose positron emission tomography (PET) demonstrated progressive LV dilatation with worsening LV systolic function, extensive non-ischaemic fibrosis, and intermittent low-grade inflammation.

In June 2024, a definite diagnosis of cardiac sarcoidosis was established after discussion at the Sarcoidosis Multidisciplinary Team meeting. Immunosuppression (intravenous methylprednisolone followed by oral prednisolone and methotrexate) achieved partial disease control; however, recurrent ventricular tachycardia persisted despite antiarrhythmic therapy. A PET scan in May 2025 showed stable scar burden with subtle new fluorodeoxyglucose (FDG) uptake in the mid-inferolateral and inferoseptal walls. Given ongoing ventricular tachycardia (VT) and biventricular dysfunction, he was referred for VT ablation and heart transplant assessment.

**Discussion:**

This case highlights the role of CMR and PET in monitoring CS, differentiating inflammatory from scar-mediated arrhythmias and guiding advanced interventions.

Learning points

- Serial multimodality imaging is essential in cardiac sarcoidosis (CS) to differentiate active inflammation from fixed scar and guide both immunosuppression and arrhythmia interventions.
- Extensive late gadolinium enhancement (LGE), even in the absence of inflammation, is a strong predictor of ventricular arrhythmias and supports proactive arrhythmia management strategies.
- Catheter ablation can be considered in cardiac sarcoidosis (CS) patients with scar-mediated ventricular tachycardia (VT) who remain symptomatic despite guideline-directed medical therapy (GDMT) and device protection.

## Introduction

Cardiac sarcoidosis (CS), a granulomatous inflammatory condition affecting 20%–30% of patients with systemic sarcoidosis,^1^ may be associated with a high risk of malignant ventricular arrhythmias (VAs) and progressive heart failure (HF).^2^ The interplay between myocardial inflammation and fibrosis creates dynamic arrhythmia substrates. Multimodality imaging, particularly the advanced modalities of cardiac magnetic resonance (CMR) and ^18^F-fluorodeoxyglucose positron emission tomography (^18^F-FDG PET), allows detection of inflammation, quantification of scar burden, and longitudinal monitoring. Management includes immunosuppression for active inflammation, device therapy for sudden cardiac death (SCD) prevention, catheter ablation for scar-mediated ventricular tachycardia (VT), and guideline-directed medical therapy (GDMT) for HF. We present a case of advanced CS followed over 3 years, where evolving imaging findings informed escalation from medical therapy with antiarrhythmics to VT ablation and advanced HF assessment as shown in the summary figure.

## Summary figure

A&E, accident and emergency; Ax, assessment; AF, atrial fibrillation; AFL, atrial flutter;, ATP, anti-tachycardia pacing; BB, beta-blocker; CAG, coronary angiogram; CMR, cardiac magnetic resonance; CRT-D, cardiac resynchronization therapy with defibrillator; CS, cardiac sarcoidosis; CTCA, computed tomography coronary angiography; CTI, cavotricuspid isthmus ablation; Dx, diagnosis; DCM, dilated cardiomyopathy; ECG, electrocardiogram; ^18^F-FDG PET, ^18^F-fluorodeoxyglucose positron emission tomography; HF, heart failure; LAD, left anterior descending artery; LFT, lung function test; LV, left ventricle; LVEF, left ventricular ejection fraction; MDT, multidisciplinary meeting; OOHVFA, out-of-hospital ventricular fibrillation arrest; SCAD, spontaneous coronary artery dissection; SCD, sudden cardiac death; hs-TnI, high-sensitivity troponin I; TTE, transthoracic echocardiogram; VT, ventricular tachycardia.

**Figure:**
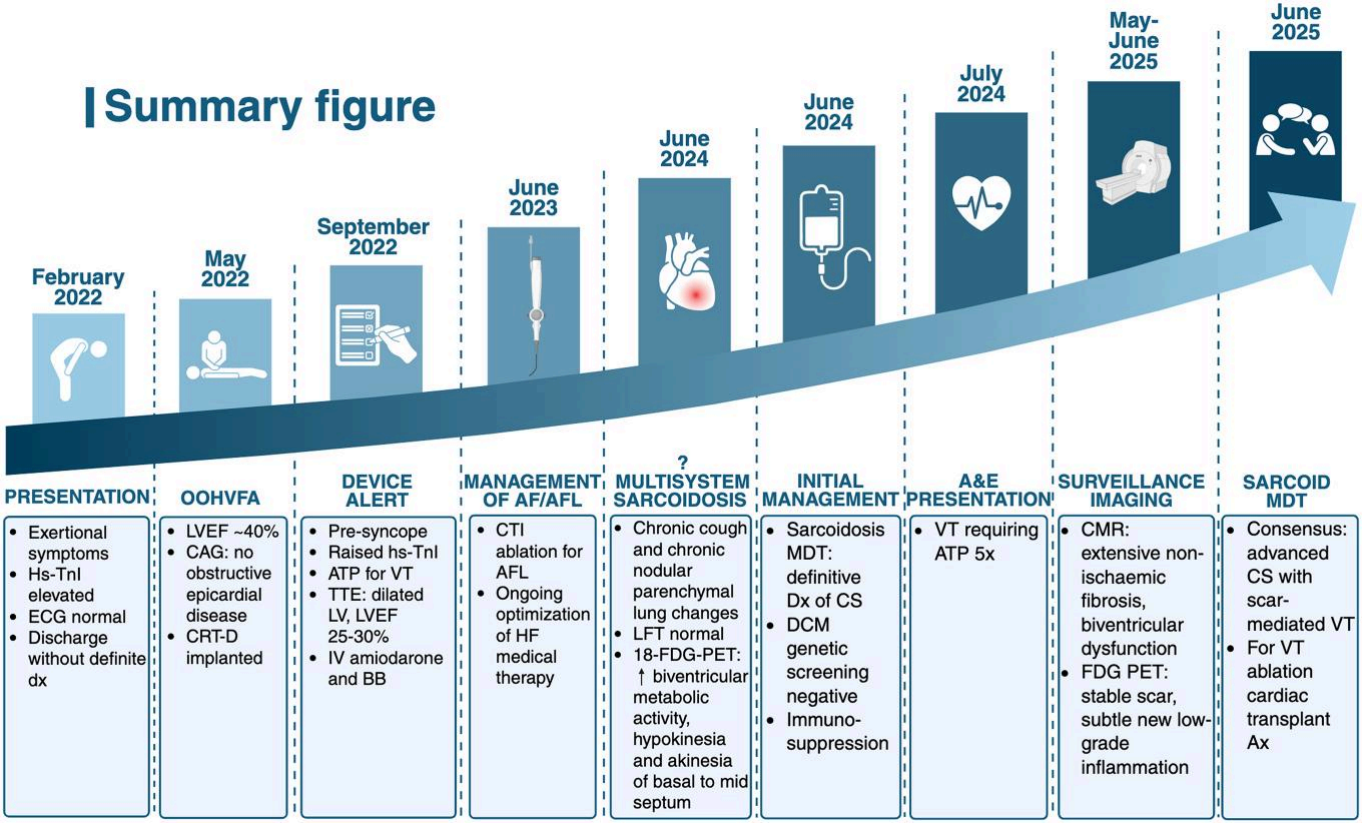


## Case presentation

A 53-year-old male with no significant cardiac history presented to his local hospital in February 2022 with exertional dizziness, tachycardia (heart rate ∼200 b.p.m.), and chest pain. Troponin was elevated, but no diagnostic electrocardiograms (ECGs).

In May 2022, he suffered an out-of-hospital cardiac arrest (OHCA) due to ventricular fibrillation (VF) after completing a 5 km run. Bystander cardiac pulmonary resuscitation (CPR) was provided. Electrocardiogram revealed new left bundle branch block (LBBB), transthoracic echocardiography (TTE) showed moderate left ventricular (LV) systolic impairment with an ejection fraction (EF) of 40%, and he underwent cardiac resynchronization therapy with defibrillator (CRT-D) for secondary prevention of SCD. Coronary angiography suggested a possible distal left anterior descending (LAD) artery spontaneous coronary artery dissection (SCAD), though findings were inconclusive. On CMR, findings were suggestive of non-ischaemic dilated cardiomyopathy (DCM).

Device interrogations over the following year detected episodes of atrial flutter, which was successfully ablated with a cavotricuspid isthmus (CTI) ablation in June 2023. In June 2024, a cardiac PET demonstrated active myocardial inflammation; lung imaging revealed parenchymal scarring consistent with sarcoidosis. Angiotensin-converting enzyme levels were normal off treatment. He received three pulses of intravenous methylprednisolone (750 mg, 1 g, 1 g), followed by oral prednisolone 30 mg once a day (suggestion of weaning by 5 mg every 2 weeks to a maintenance dose of 10 mg) and methotrexate 15 mg weekly.

**Figure 1:**
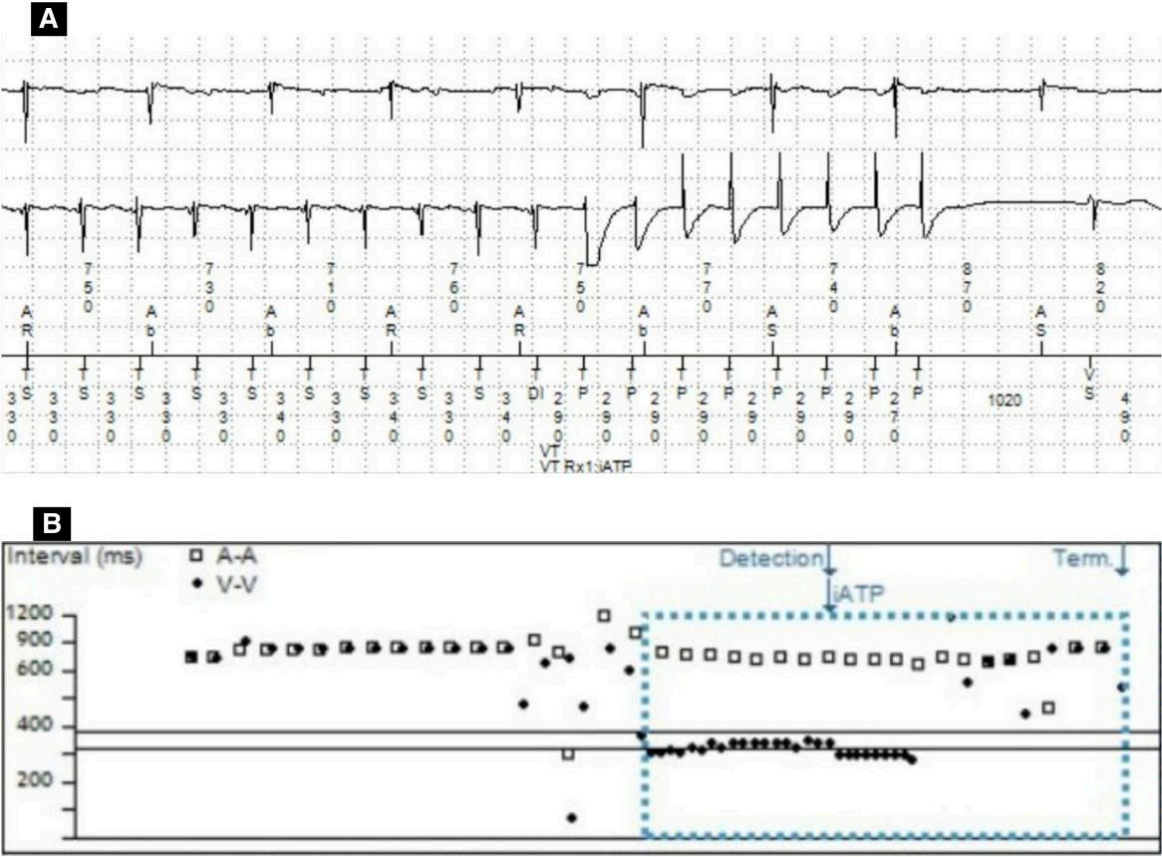
Electrogram demonstrating sustained ventricular tachycardia that required anti-tachycardia pacing. (*A*) An atrial electrogram above ventricular electrogram, showing a greater number of a ventricular sensed beat compared with atrial beats confirming a diagnosis of ventricular tachycardia at a cycle length of 330–340 ms. This is confirmed with the annotation tachycardia detected, and this is followed by eight pulses of anti-tachycardia pacing denoted by tachycardia pacing. This terminates the tachycardia to sinus rhythm as confirmed by an atrial sensed beat followed by a ventricular sensed beat. (*B*) A dot plot showing a sudden acceleration of the ventricular rate seen as the circles with maintenance of a similar atrial rate (squares), followed by anti-tachycardia pacing and resumption of sinus rhythm. EGM, electrogram; VT, ventricular tachycardia; ATP, anti-tachycardia pacing; VS, ventricular sensed beat; TD, tachycardia detected; TP, tachycardia pacing; AS, atrial sensed beat.

In July 2024, he was admitted following episodes of sustained VT that required anti-tachycardia pacing (ATP) therapy as displayed in *Figure 1*. Amiodarone was commenced. Further up-titration of bisoprolol was limited by hypotension. Transthoracic echocardiography at that time demonstrated a dilated LV with an EF of ∼25% and preserved right ventricular (RV) function.

**Figure 2:**
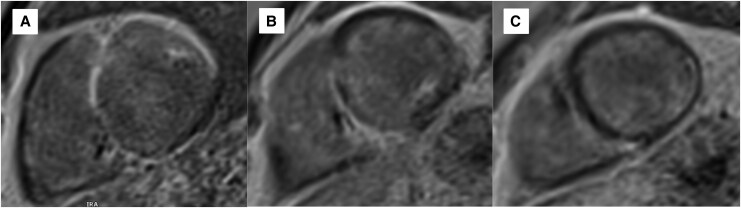
Cardiac magnetic resonance in June 2025 illustrating extensive near transmural myocardial scar, mostly subendocardium sparing, in the basal/mid-anterior, anterolateral, septal and inferior walls and partially extending into the apical lateral wall. There is fibrosis of the right ventricle inferior and lateral walls. (*A*) Short-axis base view, (*B*) short-axis mid-view, and (*C*) short-axis apical view. These findings support a diagnosis of advanced cardiac sarcoidosis with extensive myocardial fibrosis. CMR, cardiac magnetic resonance; SAX, short-axis.

Serial CMR in February 2024 and June 2025 demonstrated severely dilated LV with a left ventricular ejection fraction (LVEF) of 31%, regional wall motion abnormalities (RWMAs), and extensive non-ischaemic late gadolinium enhancement (LGE) sparing the subendocardium. The RV function was RVEF 37%. Scar involved the basal–mid-anterior, anterolateral, septal, and inferior LV walls, extending into the apical lateral wall and RV septum. No interval progression of fibrosis was evident compared to 2024 imaging. *Figure 2* shows the typical pattern of involvement, predominantly affecting the LV, particularly the free wall and interventricular septum, with the basal septum most frequently involved. Right ventricular involvement may occur but is less common. Other potential sites include the papillary muscles and atrial walls.

**Figure 3:**
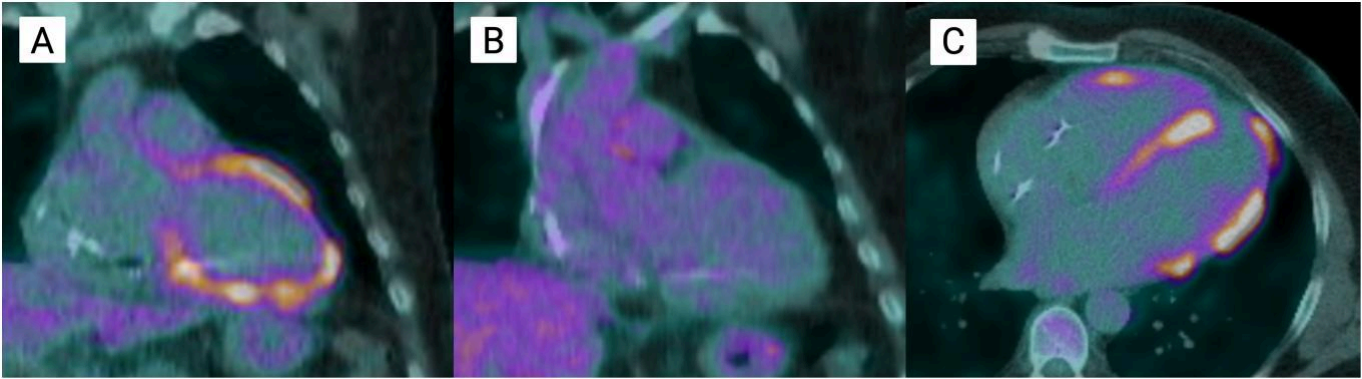
Fluorodeoxyglucose changes in response to immunosuppression. (*A*) In May 2024, fluorodeoxyglucose positron emission tomography computed tomography showed increased metabolic activity in nearly the entire left ventricle and part of the right ventricle, in keeping in active sarcoid related inflammation. (*B*) In November 2024, following treatment with high-dose intravenous glucocorticoids, repeat imaging showed complete response to treatment without increased fluorodeoxyglucose activity in the heart. (*C*) There is new low-grade increased fluorodeoxyglucose uptake in the mid-inferolateral wall and mid-inferoseptal wall, which may represent recurrence of low-grade cardiac inflammation. FDG PET, fluorodeoxyglucose positron emission tomography; CT, computed tomography.

Fluorodeoxyglucose positron emission tomography in May 2025 showed stable scar burden but new low-grade FDG uptake in the mid-inferolateral and mid-inferoseptal walls, raising the possibility of recurrent inflammation vs. incomplete suppression. No extracardiac activity was noted. *Figure 3* represents the FDG PET-CT prior to intensive immunosuppression, demonstrating subsequent resolution of inflammation, followed by low-grade recurrence of inflammation.

There was a family history of SCD in his grandparents with no definitive diagnosis. Molecular genetic testing, including next-generation DNA sequencing and analysis of a 48-gene sub-panel associated with dilated and arrhythmogenic cardiomyopathy, revealed no pathogenic or likely pathogenic variants.

**Figure 4:**
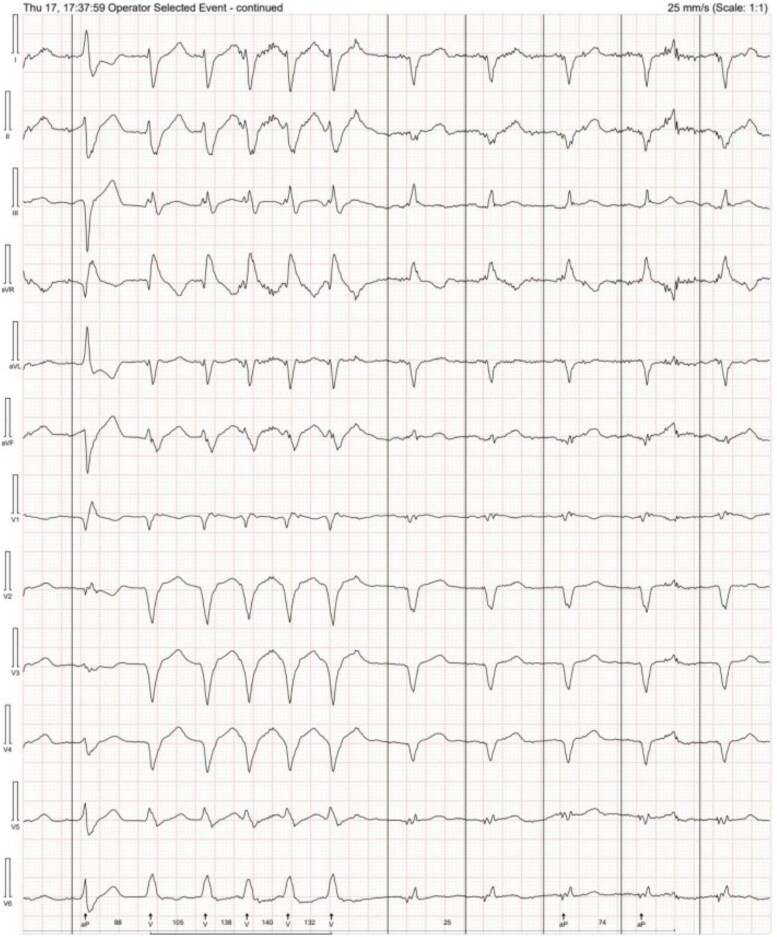
A 12-lead Holter can be a valuable tool in characterizing ventricular tachycardia morphology, specifically for identifying potential ablation targets and guiding ablation therapy. VT, ventricular tachycardia.

Due to recurrent VT despite antiarrhythmics and extensive scar without significant active inflammation, the multidisciplinary CS and electrophysiology teams recommended referral for VT ablation. A 12-lead Holter monitoring to assess ventricular ectopy (VE) and VT morphology can be reviewed in *Figure 4*. Due to progressive LV impairment and recurrent VT, the patient was also referred for transplant assessment.

## Discussion

This case of advanced CS demonstrates the complex interplay of diagnostic challenges, multimodality imaging, and evolving therapeutic strategies in managing a disease with significant arrhythmic and HF risks. The patient's journey underscores the critical role of longitudinal monitoring and multidisciplinary care in CS.^3^

The diagnostic delay observed in this case reflects a common challenge in the disease's early recognition. As noted by Ahmed *et al*. (2024),^4^ late diagnosis of CS, particularly following presentations such as high-grade atrioventricular block or VAs, is associated with worse outcomes. The absence of extracardiac symptoms highlights the need for a high index of suspicion in patients with unexplained cardiac symptoms and a family history of SCD, even without pathogenic genetic variants identified on testing.

Multimodality imaging was pivotal in guiding diagnosis and management. Magnetic resonance imaging (CMR) delineated the extent of fibrosis, with LGE revealing characteristic RWMA, consistent with patterns described by Athwal *et al*. (2022).^5^ Septal involvement, a known predictor of VAs,^6^ aligned with the patient's recurrent VT episodes. ^18^F-Fluorodeoxyglucose positron emission tomography further informed management by identifying active inflammation in June 2024, prompting escalation of immunosuppressive therapy.^7^ The persistence of low-grade FDG uptake in May 2025, despite immunosuppression, raises the possibility of incomplete inflammatory suppression, a phenomenon discussed by Liu *et al*. (2025),^8^ increasing the need for further immunosuppressive optimization.

Arrhythmia management in this case evolved from GDMT and CRT-D implantation to catheter ablation for scar-mediated VT. The patient's recurrent VT, despite antiarrhythmics, underscores the limitations of pharmacological therapy in advanced CS with extensive scar burden. As described by Ezzeddine *et al*. (2022)^9^ and Papageorgiou *et al*. (2018),^10^ catheter ablation is increasingly used as an adjunctive therapy when antiarrhythmics fail to control scar-mediated VT. The decision to pursue VT ablation aligns with emerging evidence supporting earlier interventional approaches in CS, particularly when LGE extent and septal involvement indicate high arrhythmic risk.^11^ However, the patient's progressive LV dysfunction and recurrent VT necessitated referral for transplant assessment, reflecting the guarded prognosis in advanced CS.^12^

**Figure 5:**
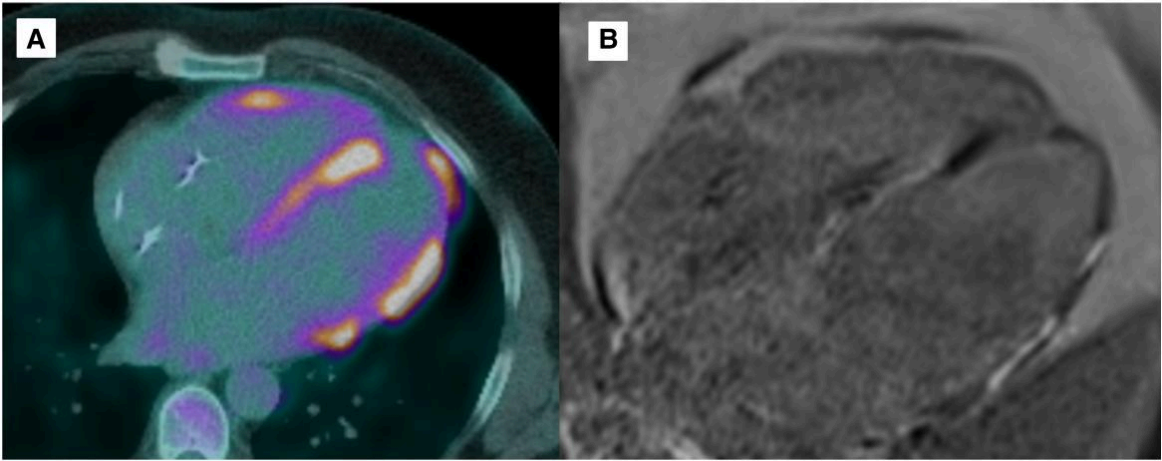
Advanced cardiac sarcoidosis with the formation of non-caseating granulomas leads to a transition from active inflammation (*A*) in the myocardium to irreversible fibrosis (*B)*. CS, cardiac sarcoidosis.

The integration of the recent European Society of Cardiology (ESC) and Heart Rhythm Society (HRS) guidelines was evident throughout the patient's care. Cardiac resynchronization therapy with defibrillator implantation following OHCA adhered to recommendations by Zeppenfeld *et al*. (2022),^13^ while imaging-guided immunosuppression and procedural interventions were consistent with the ESC consensus statement on CS by Sharma *et al*. (2024).^11^ This case highlights the shift towards scar-based risk stratification, where extensive LGE, rather than active inflammation alone, drives decisions for device therapy and ablation (*Figure 5*).

Limitations of this case include the inconclusive coronary angiography findings. Additionally, the absence of pathogenic genetic variants does not exclude a genetic predisposition, given the complex inheritance patterns in CS.^2^ Future research should focus on optimizing immunosuppressive regimens to prevent fibrosis progression and fine-tuning ablation techniques to improve outcomes in scar-mediated VT.

## Conclusion

This case exemplifies the dynamic management of CS, where multimodality imaging informs immunosuppression and risk stratification, while persistent arrhythmias warrant advanced interventions, such as VT ablation. It highlights the importance of early diagnosis, integration of the findings of advanced cardiac imaging with an MDT approach, and adherence to GDMT to mitigate the high arrhythmic and HF risks in this condition.

## Data Availability

The data underlying this article will be shared on reasonable request to the corresponding author.

## Lead author biography

Dr Rakesh Sharma is a consultant cardiologist at the Royal Brompton Hospital, Guy’s and St Thomas’s NHS Foundation Trust. He is also an honorary senior lecturer at Imperial College and Kings College, London. Dr Sharma was the clinical lead for heart failure for 7 years, during which he developed the service into its current model of advanced HF, cardio-oncology, and CS. Dr Sharma leads the internationally recognized CS service at the Royal Brompton Hospital, now one of the largest centres worldwide. He co-chaired the 2024 European Society of Cardiology Consensus Statement on Cardiac Sarcoidosis.

## Notes

**Consent:** The patient consented to the publication of his case in the scientific literature in accordance with the COPE guidelines.

## References

[1] Tavora F, Cresswell N, Li L, Ripple M, Solomon C, Burke A. Comparison of necropsy findings in patients with sarcoidosis dying suddenly from cardiac sarcoidosis versus dying suddenly from other causes. Am J Cardiol 2009;104:571–577. 19660614 10.1016/j.amjcard.2009.03.068

[2] Castrichini M, Agboola K, Siontis KC, Abou Ezzeddine O, Giudicessi J, Rosenbaum A, et al Genetic testing in presumed cardiac sarcoidosis. Eur Heart J 2023;44: ehad655–1805. 10.1161/CIRCGEN.123.004099 37401491

[3] Sharma R, Kouranos V, Cooper LT, Metra M, Ristic A, Heidecker B, et al Management of cardiac sarcoidosis: a clinical consensus statement of the Heart Failure Association, the European Association of Cardiovascular Imaging, the ESC Working Group on Myocardial & Pericardial Diseases, and the European Heart Rhythm Association of the ESC. Eur Heart J 2024;45:2697–2726.38923509

[4] Ahmed R, Sharma R, Chahal CAA. Trends and disparities around cardiovascular mortality in sarcoidosis: does big data have the answers? J Am Heart Assoc 2024;13:e034073. 38533935 10.1161/JAHA.124.034073 PMC11179766

[5] Athwal PSS, Chhikara S, Ismail MF, Ismail K, Ogugua FM, Kazmirczak F, et al Cardiovascular magnetic resonance imaging phenotypes and long-term outcomes in patients with suspected cardiac sarcoidosis. JAMA Cardiol 2022;7:1057–1066. 36103165 10.1001/jamacardio.2022.2981 PMC9475438

[6] Mathijssen H, Bawaskar PH, Rochlani Y, Georgy I, Athwal PSS, Guo Y, et al Prediction of ventricular arrhythmic outcomes in suspected cardiac sarcoidosis: a comparison of cardiovascular magnetic resonance phenotyping vs. societal recommendations for implantable cardioverter-defibrillator placement. Eur Heart J 2025;46:3583–3596. 40400457 10.1093/eurheartj/ehaf338 PMC12450518

[7] Okafor J, Azzu A, Ahmed R, Ohri S, Wechalekar K, Wells AU, et al Prognostic value of multimodality imaging in the contemporary management of cardiac sarcoidosis. Open Heart 2024;11:e002989. 39462525 10.1136/openhrt-2024-002989 PMC11529682

[8] Liu A, Munemo LT, Martins N, Kouranos V, Wells AU, Sharma RK, et al Assessment of cardiac sarcoidosis with PET/CT. J Nucl Med Technol 2025;53:123–129. 39909577 10.2967/jnmt.124.268142

[9] Ezzeddine FM, Tan N, Siontis KC. Evaluation and catheter ablation of ventricular arrhythmias in cardiac sarcoidosis. J Clin Med 2022;11:6718. 36431195 10.3390/jcm11226718 PMC9694385

[10] Papageorgiou N, Providência R, Bronis K, Dechering DG, Srinivasan N, Eckardt L, et al Catheter ablation for ventricular tachycardia in patients with cardiac sarcoidosis: a systematic review. Europace 2018;20:682–691. 28444174 10.1093/europace/eux077

[11] Sharma R, Kouranos V, Cooper LT, Metra M, Ristic A, Heidecker B, et al Management of cardiac sarcoidosis A clinical consensus statement of the Heart Failure Association. The European Association of Cardiovascular Imaging, the ESC Working Group on Myocardial & Pericardial Diseases, and the European Heart Rhythm Association of the ESC. Eur Heart J 2024;45:2697–2726.38923509

[12] Cheng RK, Kittleson MM, Beavers CJ, Birnie DH, Blankstein R, Bravo PE, et al Diagnosis and management of cardiac sarcoidosis: a scientific statement from the *American Heart Association*. Circulation 2024;149:e1197–e1216. 38634276 10.1161/CIR.0000000000001240

[13] Zeppenfeld K, Tfelt-Hansen J, de Riva M, Winkel BG, Behr ER, Blom NA, et al 2022 ESC guidelines for the management of patients with ventricular arrhythmias and the prevention of sudden cardiac death. Eur Heart J 2022;43:3997–4126. 36017572 10.1093/eurheartj/ehac262

